# Echoes of autism in family life: Neurotypical siblings’ perspectives

**DOI:** 10.1371/journal.pone.0356696

**Published:** 2026-08-26

**Authors:** Aysenur Nazik

**Affiliations:** Department of Special Education, Faculty of Education, Bartin University, Bartin, Türkiye; University of Perugia: Universita degli Studi di Perugia, ITALY

## Abstract

**Background:**

Autism spectrum disorder impacts not only the diagnosed individual but also their family members, particularly neurotypical siblings. While international research on siblings of autistic individuals has expanded, studies in Türkiye remain scarce and predominantly quantitative. This study aimed to explore in depth the lived experiences of neurotypical siblings, focusing on family dynamics, sibling relationships, coping mechanisms, and social life.

**Methods:**

A qualitative approach grounded in interpretative phenomenological analysis was adopted. 9 neurotypical siblings of individuals with autism participated. Data were gathered through semi-structured interviews, recorded, transcribed verbatim, and repeatedly reviewed for familiarity. Meaningful segments were coded, grouped into categories, and synthesized into overarching themes. Direct quotations were used to capture participants’ emotional expressions, with all identifiers anonymized. As a qualitative study, no statistical analyses were conducted.

**Results:**

The analysis revealed 4 major themes and 10 sub-themes. Sibling relationships were described as complex and fluid, marked by both closeness and distance. Communication barriers, behavioral difficulties, and disruptive or aggressive actions were highlighted as challenges that strained interactions and daily routines. Participants reported heightened family responsibilities, including caregiving and protective roles, alongside parental expectations that exceeded typical sibling duties. Despite these burdens, many siblings developed coping strategies and reported personal growth, such as enhanced empathy, patience, resilience, and responsibility. In social contexts, they encountered stigma, exclusion, and misunderstanding, yet continued to seek acceptance and support.

**Conclusions:**

The experiences of neurotypical siblings of individuals with autism embody a dual reality of strain and growth. These findings underscore the need to acknowledge siblings’ support requirements and to integrate their perspectives into holistic, family-centered autism services.

## Background

Autism spectrum disorder (ASD) is a neurodevelopmental disorder characterized by deficits in social interaction and communication, along with restricted and repetitive patterns of behavior, interests, or activities [1]. In ASD, problematic and challenging behaviors, impairments in communication and interaction, and repetitive motor movements that affect daily life may vary from one diagnosed individual to another [1]. Individuals diagnosed with ASD may experience difficulties making and maintaining friendships, communicating with others, or understanding what behaviors are expected of them in social settings, including school or work environments [2,3]. According to recent studies, while the World Health Organization (WHO) [4] reports that autism is observed in approximately 1 out of every 100 children worldwide, data from the Autism and Developmental Disabilities Monitoring Network of the United States Centers for Disease Control and Prevention (CDC) indicate that the prevalence of autism was reported as 1 in 150 in 2000–2002, 1 in 110 in 2006, 1 in 68 in 2010, 1 in 59 in 2014, and 1 in 31 in 2022 [5].

### Experiences of the neurotypical sibling of ASD

Autism affects not only the child who has received the diagnosis, but also the family members living with that child because of the conditions it involves [6,7]. Caring for a family member with ASD leads all family members to experience different psychological states [8–11]. Having an individual with ASD in the family causes all family members to need more help in terms of social and emotional support and access to information [12]. The presence of an individual with ASD in the family affects not only the primary caregivers, the mother and father, but also the typically developing siblings in the family [13–20].

Studies conducted with siblings of individuals diagnosed with different disabilities have reported that those who have a sibling with ASD experience more intense stress and have less social communication and interaction with their siblings [21–23]. In addition, living with a sibling with ASD may cause the typically developing sibling to feel less loved, rejected, ignored, or forced to feel ashamed [14,24–26]. It has also been found that when a sibling is diagnosed with ASD, this situation creates a form of negative stigma for typically developing siblings as well, and that together with this stigma, they experience more intense feelings such as depression, shame, and avoidance [18,27–29]. There are also studies in the literature showing that, due to the challenging experiences of living with siblings with ASD, the social relationships of typically developing siblings [30,31], as well as their experiences of making and maintaining friendships [32], are negatively affected.

Some studies have shown that sibling relationships change and develop over time, and that this change can provide an opportunity to build positive relationships between siblings with ASD and their typically developing siblings [8,27,28,33]. Some siblings have stated that growing up with a sibling with ASD not only contributes to a better understanding of autism, but also fosters positive feelings such as empathy, patience, kindness, supportiveness, and love, and has a positive impact on their lives [34–38]. In studies conducted with typically developing siblings, siblings reported that they feel a heavy sense of responsibility in helping and caring for their siblings with ASD [39–41]. They also stated that, especially at first, they experienced various difficulties in communicating with their siblings with ASD, and that this situation sometimes led to frustration and unhappiness; however, over time, these feelings were replaced by feelings such as protectiveness and love [14,26,40]. Social support provided by family and the surrounding environment plays a very important role in coping with the behavioral and emotional problems frequently seen in individuals with ASD [42]. From the perspective of neurotypical siblings (NTS), it has likewise been found that when age-appropriate information and support are provided, siblings can establish more constructive relationships with their siblings with ASD [43].

When studies conducted over more than fifteen years are examined, it is seen that research focusing on the typically developing siblings of individuals with ASD has increased [44–49]. There are several reasons for this. First, studies on sibling relationships show that the relationship between siblings has an important function in psychosocial development [50]. In this context, growing up with a sibling with developmental impairments in communication and social interaction is thought to affect a child’s social and emotional development. In addition, siblings are known to be among those who spend the most time with the individual with ASD after the parents. For this reason, the sibling’s disability is likely to have important effects on the well-being and adjustment processes of typically developing siblings.

In addition to all these reasons, researchers working on sibling-mediated interventions for children with ASD [51,52] emphasize that understanding the psychosocial functioning of typically developing siblings is important for shaping and individualizing future sibling-mediated intervention programs [53]. The great majority of studies conducted with typically developing siblings of children with autism have been carried out using quantitative methods [15,18]. Although studies using quantitative research methods have accurately described the measurable effects of ASD on siblings, they have nevertheless been insufficient to reveal which variables influence these effects in detail. Research concerning siblings of individuals with ASD has generally been conducted with parents or caregivers who are primarily responsible for the care of the child with ASD [12,54], and moreover, the majority of these studies have addressed the issue only from the perspectives of mothers [55,56]. In Turkey, the literature on siblings of individuals with ASD has predominantly relied on quantitative research methods [57–61]. Accordingly, the present study aims to contribute to the Turkish literature by offering an in-depth examination of the perspectives of NT siblings of individuals with ASD.

## Methods

### Research design

Interpretative Phenomenological Analysis (IPA) was selected as the method of data analysis in this research. IPA aims to provide a detailed understanding of participants’ lived experiences by focusing on the major themes that arise from their narratives. From this perspective, participants are treated as experts in relation to their own personal worlds [62]. In this study, IPA was used to explore how NT siblings perceive and interpret the experience of having a sibling with ASD.

This study is reported in accordance with the Consolidated Criteria for Reporting Qualitative Research (COREQ), a 32-item checklist developed to promote transparent and complete reporting of qualitative studies based on interviews and focus groups [63].

### Participants

In IPA studies, the number of participants is intentionally limited so that each participant’s lived experience can be examined in depth. Rather than collecting superficial data from a large number of individuals, this approach focuses on developing a comprehensive understanding of experiences related to a particular phenomenon and the meanings attributed to those experiences [64].

The study participants were selected through criterion sampling, a type of purposive sampling. Criterion sampling is considered particularly useful in qualitative research for addressing the research problem effectively [65]. The inclusion criteria were as follows: (a) being between 8 and 18 years of age, (b) having no formally identified diagnosis of special needs, (c) living in the same household with a sibling diagnosed with ASD, and (d) volunteering to participate in the study. A total of nine neurotypical (NT) siblings who met these criteria and agreed to participate were included in the study.

The age range of 8–18 years was adopted because children and adolescents within this developmental period are generally able to verbally reflect on their experiences and articulate their perceptions of the sibling relationship, while typically still sharing the family home with their sibling with ASD. The criterion of having formally identified diagnosis of special needs referred to NT siblings who had no formally identified developmental, intellectual, psychiatric, or other special education diagnosis. This information was based on parental report obtained during recruitment rather than on an independent clinical or professional screening; this is acknowledged as a limitation of the study. Living with a sibling diagnosed with ASD was defined as having continuously shared the same household with the sibling with ASD since the sibling’s birth, thereby ensuring sustained daily contact. Participants were recruited from among NT siblings who had at least one sibling with autism attending rehabilitation centers in the central district of Bartın province. Families of children with ASD were reached through these rehabilitation centers and were informed about the study. The researcher then contacted siblings from families who met the inclusion criteria and volunteered. The siblings with ASD were identified in community-based rehabilitation settings rather than in inpatient or residential institutions. Written informed consent was obtained from the NT siblings who agreed to participate in the study, and written parental consent was obtained from the families of participants under 18 (n = 8). The neurotypical siblings were coded as NTS1, NTS2, …, NTS9. All demographic information is presented in Table 1.

**Table 1:** Demographic profiles of siblings with and without ASD.

Participants	Age of NTS	Gender of NTS	Age of ASDS	Gender of ASDS
NTS 1	17	Female	9	Female
NTS 2	11	Male	6	Male
NTS 3	15	Male	19	Male
NTS 4	16	Female	18	Female
NTS 5	9	Male	12	Female
NTS 6	12	Female	15	Male
NTS 7	10	Female	3	Male
NTS 8	13	Male	10	Female
NTS 9	18	Female	15	Female

*Note.* NTS: Neurotypical Sibling, ASDS: Autism Spectrum Disorders Sibling.

### Data collections

The research data were collected between October 13 and 23, 2025, using a semi-structured interview form, following approval from the ethics committee. Before each interview, the researcher conducted a brief introductory conversation to help the participant become familiar with the setting and to feel comfortable. During the development of the interview questions, the opinions of four academics were consulted: two specializing in special education and two in psychology. The first question was designed to allow participants to express their experiences freely and at their own pace, while the subsequent questions aimed to elicit more detailed descriptions of their lived experiences. The interview form collected general information about the participant and their family, the relationship with the sibling with ASD, the positive aspects of having a sibling with ASD, the difficulties encountered in daily life, and coping strategies and sources of support. The interviews were conducted in the participants’ homes and ranged from 23 to 48 minutes, with an average of 39 minutes.

### Procedure

Informed consent procedures were implemented in accordance with each participant’s age. For the eight participants who were younger than 18 years, written informed consent was obtained from a parent or legal guardian, together with written assent from the participant. For the 18-year-old participant (NTS 9), written informed consent was obtained directly from the participant. Throughout data collection, the researcher repeatedly reminded participants that their participation was voluntary and that they could withdraw at any time without any consequences. Data were collected without personal identifiers, and transcripts were stored for analysis on a password-protected computer accessible only to the researcher. After each interview, the recording was assigned a participant number, uploaded as an encrypted file, and subsequently deleted from the recording device.

### Data analysis

The researcher listened repeatedly to all interviews with the NT siblings to gain familiarity and transcribed using Microsoft Word Dictate. The transcribed interviews were then reviewed line by line to determine whether any points had been overlooked. The researcher examined printed copies of the transcribed recordings and developed preliminary themes for each interview. This process was repeated for each NT sibling until all recordings had been completed.

Using Microsoft Word, the researcher coded each participant’s responses by color highlighting text segments representing meaningful units. Codes reflecting similar content were then grouped under broader categories, and these categories were further synthesized into themes.

These procedures followed the established analytic steps of interpretative phenomenological analysis [62]. First, each transcript was read and re-read to achieve immersion in the participant’s account. Second, initial exploratory notes were made on the descriptive, linguistic, and conceptual features of the data. Third, these notes were developed into emergent themes for each individual case. Fourth, connections between emergent themes were examined and clustered into higher-order (superordinate) themes at the level of the individual case. Fifth, this idiographic process was repeated for each participant in turn, with care taken to bracket insights from earlier cases before analyzing the next. Finally, patterns were examined across all cases to develop the final set of superordinate’s themes and subthemes reported here. Throughout, the analysis moved iteratively between the parts and the whole of the data, in line with the hermeneutic circle, and each interpretation was checked against participants’ own words to ensure it remained grounded in the data.

At the end of this process, a final thematic structure consisting of 4 main themes and 10 sub-themes was developed by combining similar themes identified across different data sets. To preserve the emotional expressions embedded in the interviews, the statements that formed these themes were presented as direct quotations. To maintain confidentiality, all names mentioned in the interviews were replaced with codes.

### Credibility & confirmability

Credibility and confirmability of the data were ensured through several methodological strategies [65]. The author who conducted the study has 10 years’ experience in the field of special education and has worked extensively with individuals with ASD and their families. This professional background is acknowledged, in line with researcher reflexivity, as potentially shaping the author’s assumptions, perspectives, and interpretations regarding sibling relationships in families in which one sibling has an autism diagnosis. As a measure to support the reliability of transcripts, a randomly selected subset of interviews representing at least 30% of the dataset (n = 3) was submitted to an expert with experience in interviewing for verification of transcription accuracy, consistent with external audit procedures. Consistent with thick description, all analyses were grounded in the study findings, and direct quotations were used to illustrate experiential themes, which were based on the siblings’ unique accounts and the researchers’ interpretations of those experiences.

Reflexivity was maintained throughout the research process. The author kept a reflexive journal during data collection and analysis to record assumptions, emotional responses, and interpretive decisions, and to remain aware of how her professional background as a special educator might shape the analysis. Although IPA is an idiographic and interpretative approach in which the analysis is commonly conducted by a single analyst, methodological rigour was supported through several strategies: independent verification of transcription accuracy for a subset of interviews by an external expert (as described above); the maintenance of an audit trail linking raw data, codes, categories, and themes; and repeated reflexive review of the developing thematic structure against the original transcripts [62,64]. No formal independent double-coding or member checking was undertaken; this is acknowledged as a limitation.

## Results

As a result of the analysis of the data obtained from the semi-structured interviews, 4 main themes and 10 sub-themes were identified. The main themes were responsibility and expectations; navigating between closeness and distance in the sibling relationship; learning to cope with challenges and adapt; and navigating social life. The main categories, sub-categories and representative codes are presented in Table 2.

**Table 2:** The main categories, sub-categories and representative codes.

Main themes	Sub-themes	Representative codes
Responsibility and expectations	Assuming responsibility for the sibling and undertaking caregiving	Doing household and caregiving tasks alone; parentified role; the sibling’s needs placed before one’s own
	Increasing expectations	Parental pressure to be mature and to be the older one; deprioritizing own needs; feeling unheard
	Assuming a protective role toward the sibling	Shielding the sibling from others; defending against ridicule; vigilance in public settings
Navigating between closeness and distance in the sibling relationship	Feelings of love, closeness, and attachment	Strong sibling bond; affection despite differences; wish for reciprocal connection
	Challenges and distance in the relationship	Separate worlds; limited shared time; conflict, misunderstanding, and withdrawal
	Efforts to maintain the sibling bond	Persisting despite frustration; accepting the sibling as they are; adjusting expectations
Learning to cope with challenges and adapt	The effects of problem behaviors on daily life	Public embarrassment; disrupted plans and routines; stress and worry
	Fostering emotional and social adaptation	Acceptance over time; reframing family difference; reduced anger and personal growth
Navigating social life	Experiences of judgment, labeling, and misunderstanding	Stigmatizing stares and whispers; mislabeling as “spoiled”; anger and sadness
	Seeking acceptance and social support	Desire to be treated normally; wish for understanding; relief when accepted

### Main theme 1: Responsibility and expectations

Some of the NT siblings stated that family roles were distributed unfairly, and that their parents expected them to take on more responsibility and, in many cases, to behave as adults rather than as children. Most of the NT siblings reported feeling responsible for protecting their siblings from the surrounding environment and other people.

### Sub-theme 1.1 Assuming responsibility for the sibling and undertaking caregiving

*“Most of the time, I have to do things on my own because my mom and dad are always busy with my brother”* (NTS 6).

*“They always expect me to act like I’m older than I am; especially my mom—she tells me to ‘deal with your brother, that’s not normal’ and always does whatever he wants”* (NTS 2).

### Sub-theme 1.2: Increasing expectations

*“Even when I was little, my mother often told me phrases such as ‘You’re the smart one,’ ‘Take care of your sibling,’ or ‘You’re the older one.’ I heard those phrases so often that over time I* *learned to deprioritize my own needs. Everyone at home thought my sibling needed more support. They might be right, but I wish they’d ask me how I felt too”* (NTS 9).

*“My parents (my mother and father) always expect me to back down. Whenever there’s a problem at home, I’m usually the one who steps back. I’m the one who gives in, demonstrates patience, and tries to keep the peace to avoid arguments. I’m very dissatisfied with this situation. Sometimes I want to be understood too”* (NTS 5).

### Sub-theme 1.3. Assuming a protective role toward the sibling

*“I believe the urge to protect my sibling developed within me over time. I learned how to protect him. When I was little, I thought I was helping him, but over time I realized I was trying to protect him from people outside our family. People are not being understanding toward him. That’s why, when I’m by his side, it feels like no one could ever hurt him”* (NTS 1).

*“Our schools are on the same campus, and the buildings are adjacent. It really bothers me, especially during recess, when someone says something mean to my brother or acts as if they’re making fun of him. It feels as if it’s being done to me. That’s why I want to intervene immediately and defend him. Because he can’t protect or defend himself”* (NTS 8).

### Main theme 2. Navigating between closeness and distance in the sibling relationship

This theme shows that having a sibling with autism is experienced as a layered, complex relationship, marked by a tension between a desire for emotional closeness and a need for some distance. Participants spoke about feelings of love, attachment, and affection toward their siblings, while also describing the communication challenges, emotional strain, and occasional sense of separation that can accompany autism. Despite these difficulties, participants developed different ways to preserve and strengthen their relationships with their siblings.

### Sub-theme 2.1. Feelings of love, closeness, and attachment

*“Maybe I can’t have long, deep conversations with her as I do with my other siblings, but having an older sister, “even if she has autism”, makes me feel good. We don’t have a typical* *sibling relationship, but I still think there’s a strong bond between us. Because she’s my sister, and I love her very much”* (NTS 4).

*“My sibling is still a baby; maybe she doesn’t know how to play yet. I always approach her and offer her toys, so she’ll include me in her play. I want her to love me when she grows up”* (NTS 7).

### Sub-theme 2.2. Challenges and distance in the relationship

*“He’s older than me, but he acts like a child. That’s why we didn’t spend much time together, especially when we were both young. He has his own world, nevertheless. He doesn’t act very close to me”* (NTS 4).

*“We fight a lot when we’re together. He can’t express himself properly because he can’t say what he wants; when I don’t understand him, he becomes angry and yells. Instead of fighting, I prefer to go to my room and be alone”* (NTS 8).

### Sub-theme 2.3. Efforts to maintain the sibling bond

*“Sometimes I become very angry with him and I even tell myself, ‘That’s it, I’m not going to bother with him anymore; let him figure it out on his own.’ However, I can’t bring myself to do it and return to him anyway. Because we don’t have any other siblings neither he nor I”* (NTS 5).

*“I recognize that I must build a relationship with him without expecting anything from him, because he has autism.Whenever I set expectations, it unfortunately always ends in disappointment. I’ve learned to accept him just as he is. This is less exhausting for both of us”* (NTS 9).

### Main theme 3. Learning to cope with challenges and adapt

Within this theme, the NT siblings stated that, over time, they learned to cope with the difficulties they encountered and developed various strategies to adapt to the situation. Although they reported that their siblings’ problem behaviors and the difficulties these behaviors created in daily life were challenging for them, they also expressed that, over time, they developed different ways of coping with these situations.

### Sub-theme 3.1. The effects of problem behaviors on daily life

*“Sometimes he suddenly begins to scream or throw himself onto the ground. He engaged in this behavior more during childhood; now he does so less. I used to become highly stressed when we were outside among other people I felt embarrassed. On the one hand, I feel bad for my brother; I just want him to calm down and get back to normal as soon as possible”* (NTS 3).

*“Occasionally, we cannot go where we want because of my brother’s behavior. For example, there have been many occasions when we were about to leave the house but changed our minds and returned. It is as if we have to plan everything around him. At the end of every plan, we wonder, ‘Will there be a problem?’”* (NTS 6).

### Sub-theme 3.2. Fostering emotional and social adaptation

*“I didn’t understand much when I was a child. I even thought my mom and dad loved him more and pushed me to the sidelines. Because he has autism, they provided him with increased attention. Then, as I got older, I learned that I had to accept this situation and reshape my life as if I had a sibling with autism”* (NTS 9).

*“It took me some time to accept certain things about my brother, to be honest. W hen I was little, I was often distressed, wondering why everything in our house was so different. However, I now see things differently. Every family has its own struggles; this is ours. Once I accepted that, the anger inside me subsided a bit”* (NTS 1).

### Main theme 4. Navigating social life

This theme highlights that participants’ experiences of living in society alongside their siblings who have autism were largely influenced by others’ attitudes and the nature of their social interactions. It suggests that experiences of exclusion, along with efforts to gain acceptance and to access social support, were closely intertwined in shaping participants’ experiences.

### Sub-theme 4.1. Experiences of judgment, labeling, and misunderstanding

*“Individuals often form judgments without knowing the facts. They label my sister’s behavior as spoiled or ill-mannered without understanding the reason behind it. It makes me both very angry and very sad”* (NTS 4).

*“I’m so tired of people staring at us and pointing us out to each other. There have been times when I did not even want to go to the grocery store with my sister just because of this. When people see my brother, they immediately start whispering. This really bothers me”* (NTS 1).

### Sub-theme 4.2. Seeking acceptance and social support

*“Maybe not everyone has to understand everything, but I at least want them to be a little understanding. Because sometimes it feels good just to be treated normally”* (NTS 6).

*“When they don’t look at my brother with pity, I think, ‘Finally.’ They now understand us. It’s actually that easy to be understood” (*NTS 3).

## Discussion

The present study focuses on the experiences of NT siblings of individuals diagnosed with autism spectrum disorder in Turkey, addressing the limited prior examination of this topic. Based on the analyses conducted, the themes that emerged were discussed in relation to the study’s contextual characteristics and to the existing literature.

### Responsibility and expectations

The findings of the study indicate that siblings of individuals with ASD face a range of challenges within their sibling relationships. Previous research suggests that these difficulties are influenced by multiple factors, including limited knowledge about the diagnosis, shifts in family roles and responsibilities, negative reactions from others, and the coping resources available to the family [10,28,41,49,66–69]. Further analysis of the sub-themes shows that NT siblings not only provide care for their siblings with ASD but also take on protective roles. This is in line with earlier studies reporting that typically developing siblings may at times assume caregiving responsibilities that resemble those of a parent [26,37,48,68]. Moreover, NT siblings reported that they carried greater responsibilities than their siblings with autism, both inside and outside the home, and that their parents tended to expect more from them. These results are consistent with the existing literature, which shows that typically developing siblings often shoulder greater responsibilities [70] and may sometimes adopt roles similar to that of a mother [14].

### Navigating between closeness and distance in the sibling relationship

The study’s findings suggest that NT siblings often find themselves caught between emotional closeness and emotional distance in their relationships with their siblings with ASD. Although they reported accepting their siblings’ differences, they also stated that they frequently had difficulty making sense of what those differences meant in practice. Communication limitations, tantrums, behavioral difficulties, and aggressive behaviors emerged as major factors complicating sibling interactions. This finding aligns with previous research showing that relationships with siblings with ASD are often experienced as challenging because of communication-related and behavioral difficulties [28,41,71]. Consistent with the literature, participants also noted that as they grew older, they developed a more positive understanding of their siblings’ condition [8,36,49].

Similarly, difficulties in communication and social interaction appear to be central factors shaping experiences of closeness and distance in sibling relationships. The literature indicates that children with ASD often experience marked difficulties in expressive and receptive language, as well as in the social use of language [72]. Such difficulties can restrict reciprocal interaction between siblings and may sometimes cause typically developing siblings to pull back from the relationship [14,15,73,74]. As a result, communication problems not only weaken the sibling bond but also highlight the fragile nature of the relationship.

NT siblings of individuals with autism also reported that the challenging characteristics of autism require them to put extra effort into sustaining the sibling relationship and, at times, make personal sacrifices. Likewise, previous studies have shown that communication difficulties and behavioral problems can complicate sibling interactions [14,75], yet despite these challenges, efforts to preserve the sibling relationship often continue [27,73,76].

### Learning to cope with challenges and adapt

The analyses showed that the problem behaviors of siblings with ASD had a negative impact on the daily lives of NT siblings and contributed to difficulties in communication and interaction. Previous studies similarly indicate that the behavioral problems and communication challenges associated with siblings with ASD place an emotional burden on NT siblings [36,41,77,78]. Another important finding of the study relates to the emotional and social adjustment of NT siblings. The literature suggests that NT siblings who have a sibling with a developmental disability may, over time, develop qualities such as empathy, patience, responsibility, and resilience [34,36,40,79].

### Navigating social life

The final main theme of the study indicates that NT siblings face various difficulties not only within the family but also in the broader social environment. The siblings reported exposure to negative, stigmatizing attitudes in social situations and stated that these attitudes led to feelings of shame, anger, and frustration, and at times caused them to withdraw from social settings. Similarly, the literature indicates that NT siblings of individuals with ASD may encounter stigmatizing attitudes, and that this can have emotionally and socially challenging consequences [49,68]. In particular, when the behaviors of the sibling with ASD are misinterpreted by others, these siblings may feel the need to explain both themselves and their families [27,40]. In this respect, the present findings are consistent with studies showing that siblings also struggle to be understood and accepted in the social environment [28,41].

### Limitations

The study has several limitations. First, due to the nature of interpretative phenomenological analysis, the study was conducted with a limited number of participants (n = 9). Although IPA allows for an in-depth examination of participants’ experiences, it limits the transferability of the findings to larger groups. In addition, the data were collected only through a semi-structured interview form and were not supported by observations, parent interviews, or other data collection methods. Therefore, the findings should be interpreted on the basis of the participants’ subjective accounts. Another limitation is that the study focused solely on the experiences of NT siblings.

## Conclusions

The study’s results indicate that the experiences of NT siblings of individuals diagnosed with ASD are multidimensional and dynamic. The findings indicate that NTSs are positioned within the family not only in the role of a sibling but also in roles involving increasing responsibilities, caregiving burdens, and expectations. The study also reveals that communication difficulties, behavioral problems, and limitations in social interaction associated with ASD lead to changes in their relationships with their siblings with ASD. At the same time, the findings show that having a sibling with ASD is not limited to difficulties and negative experiences for NT siblings and can also support the development of psychosocial resources such as empathy, patience, psychological resilience, and adjustment skills. The study’s findings highlight the importance of making the support needs of NT siblings visible and taking them into account in intervention processes. In this context, support services for autism should be addressed from a holistic perspective that also includes the sibling experience.
